# Gene Expression Profiling for Diagnosis of Triple-Negative Breast Cancer: A Multicenter, Retrospective Cohort Study

**DOI:** 10.3389/fonc.2019.00354

**Published:** 2019-05-07

**Authors:** Qifeng Wang, Midie Xu, Yifeng Sun, Jinying Chen, Chengshu Chen, Chenhui Qian, Yizuo Chen, Liyu Cao, Qinghua Xu, Xiang Du, Wentao Yang

**Affiliations:** ^1^Department of Pathology, Fudan University Shanghai Cancer Center, Shanghai, China; ^2^Department of Oncology, Shanghai Medical College, Fudan University, Shanghai, China; ^3^Canhelp Genomics, Hangzhou, China; ^4^Department of Thyroid and Breast Surgery, The First Affiliated Hospital of Wenzhou Medical University, Wenzhou, China; ^5^Department of Biomedical Engineering, University of California, Irvine, Irvine, CA, United States; ^6^Institute of Machine Learning and Systems Biology, College of Electronics and Information Engineering, Tongji University, Shanghai, China

**Keywords:** triple-negative breast cancer, tissue of origin, gene expression profiling, quantitative real-time PCR, tumor classification

## Abstract

**Background:** Triple-negative breast cancer (TNBC) accounts for 12–20% of all breast cancers. Diagnosis of TNBC is sometimes quite difficult based on morphological assessment and immunohistochemistry alone, particularly in the metastatic setting with no prior history of breast cancer.

**Methods:** Molecular profiling is a promising diagnostic approach that has the potential to provide an objective classification of metastatic tumors with unknown primary. In this study, performance of a novel 90-gene expression signature for determination of the site of tumor origin was evaluated in 115 TNBC samples. For each specimen, expression profiles of the 90 tumor-specific genes were analyzed, and similarity scores were obtained for each of the 21 tumor types on the test panel. Predicted tumor type was compared to the reference diagnosis to calculate accuracy. Furthermore, rank product analysis was performed to identify genes that were differentially expressed between TNBC and other tumor types.

**Results:** Analysis of the 90-gene expression signature resulted in an overall 97.4% (112/115, 95% CI: 0.92–0.99) agreement with the reference diagnosis. Among all specimens, the signature correctly classified 97.6% of TNBC from the primary site (41/42) and lymph node metastasis (41/42) and 96.8% of distant metastatic tumors (30/31). Furthermore, a list of genes, including *AZGP1, KRT19*, and *PIGR*, was identified as differentially expressed between TNBC and other tumor types, suggesting their potential use as discriminatory markers.

**Conclusion:** Our results demonstrate excellent performance of a 90-gene expression signature for identification of tumor origin in a cohort of both primary and metastatic TNBC samples. These findings show promise for use of this novel molecular assay to aid in differential diagnosis of TNBC, particularly in the metastatic setting.

## Introduction

Breast cancer is the most common malignancy and the sixth leading cause of cancer-related mortality among women in China, accounting for ~2,686,000 new cases and 695,000 deaths in 2015 (1). Histologically, breast cancer is a heterogeneous disease with distinct subtypes and pathological features, leading to variable treatment options, and prognoses. Triple-negative breast cancer (TNBC) accounts for 12–20% of all breast cancer cases and is characterized by a lack of estrogen receptor (ER) and progesterone receptor (PR) expression, combined with an absence of both overexpression and amplification of the human epidermal growth factor receptor-2 (HER-2) gene (2).

TNBC is associated with a high rate of relapse and poor outcomes within the first 3 years after treatment (3, 4). Given the latest promising data on immunotherapy, precise diagnosis of this malignancy is more important than ever for determining patient prognosis and facilitating patient-tailored therapy (5). In most cases, the primary tumor can be recognized based on morphological assessment and immunohistochemistry (IHC). Mammaglobin (MGB) and gross cystic disease fluid protein-15 (GCDFP-15) are currently the best immunohistochemical markers available for metastatic breast cancer, with reported overall sensitivities ranging from 50 to 87% and 10 to 79%, respectively (6). However, both markers demonstrate considerably lower sensitivities in TNBC than in ER-positive tumors (7–9). Therefore, clinical identification of the site of tumor origin, in particular for metastatic cancers without a prior history of breast cancer, is difficult and thus urgently needed.

In recent years, efforts have been made toward establishing new supplementary diagnostic tools for identification of primary tumor sites. Molecular profiling is a promising diagnostic approach that has the potential to provide an objective classification of metastatic cancers with an uncertain or unknown tissue of origin and to facilitate more time- and cost-effective diagnostic work-up of cancer patients (10). Molecular diagnostic profiling methods that use either microarrays or real-time reverse transcription polymerase chain reaction (RT-PCR) have been developed to classify a multitude of tumor types or to diagnose certain types of cancer. Kerr et al. described a 92-gene molecular classifier with an overall accuracy of 99% for determination of the site of origin of tumors with neuroendocrine differentiation (11). Additionally, Benjamin et al. analyzed microRNA expression profiles to identify malignant pleural mesothelioma (12).

Previously, we developed a pan-cancer gene expression signature with an overall accuracy of 97.1% for classification of carcinomas originating in 22 major tissue types, including adrenal gland, brain, breast, cervix, colorectum, endometrium, gastroesophagus, head and neck, kidney, liver, lung, lymphatic tissues, skin, mesothelial tissues, neuroendocrine tissues, ovary, pancreas, prostate, connective tissue, testis, thyroid, and urinary tract (13). Recently, we updated this gene expression-based signature by eliminating lymphoma-related genes and reference tumor samples to reduce the influence of lymphocytes when classifying lymph node metastases. Therefore, a new version of the gene expression signature was developed using 90 tumor-specific genes corresponding to 21 major tumor types (14). The aim of the current study was to evaluate the performance and highlight the potential diagnostic utility of this 90-gene expression signature for identifying the anatomical origin of TNBC tumors. In addition, exploratory analyses were conducted to examine and identify subsets of genes within the 90-gene panel for specific use in the diagnosis of TNBC.

## Materials and Methods

### Sample Selection

This study was approved by the institutional review board of Fudan University Shanghai Cancer Center; Zhejiang Cancer Hospital; The First Affiliated Hospital of Wenzhou Medical University; The First People's Hospital of Changzhou; The Third Affiliated Hospital of Soochow University; Changzhou No.2 People's Hospital; the Affiliated Hospital of Nanjing Medical University and The Affiliated Hospital of Jiangnan University. Formalin-fixed paraffin-embedded (FFPE) tissue samples from 73 breast cancer patients (42 with lymph node metastasis and 31 with distal metastasis) archived from 2014 to 2017 were used in this study. All samples were excisional biopsies and histologically confirmed as ER negative, PR negative, and HER-2 negative. Examples of HER-2 negative results determined with Fluorescence *in situ* Hybridization were shown in Figure S1. In addition, 12 non-TNBC tumor samples including four cases with lymph node metastasis and eight cases with distal metastasis were enrolled in this study. Before inclusion, hematoxylin and eosin (H&E)-stained slides from tumor samples were reviewed by pathologists for evaluation of the percentage of tumor cells and necrotic areas. If fewer than 60% tumor cells or >40% necrotic area was present by inspection, regions of interest were circled on the H&E-stained slides, and the corresponding areas from unstained FFPE tissue sections were then manually macrodissected for tumor enrichment.

### Sample Preparation and RNA Isolation

Total RNA was isolated from FFPE tissue sections using an FFPE Total RNA Isolation Kit (Canhelp Genomics, Hangzhou, China). Briefly, paraffin sections were placed in sterile 1.5-ml microcentrifuge tubes, deparaffinized with 100% xylene, and washed twice in 100% ethanol. Deparaffinized tissue was digested with proteinase K at 56°C for 15 min and then incubated at 80°C for another 15 min to partially reverse the crosslinking of nucleic acids. Samples were DNase treated and eluted in 40 μl of RNase-free water. The concentration of total RNA was spectrophotometrically determined using total absorbance at 260 nM, and purity was quantified using the A260/A280 ratio. RNA samples with A260/A280 ratios of 1.9 ± 0.2 were included in this study.

### Expression Profiling of 90 Tumor-Specific Genes

For each sample, cDNA was generated from total isolated RNA using a High-Capacity cDNA Reverse Transcription Kit with RNase Inhibitor (Applied Biosystems, Foster City, CA, United States). The expression profiles of 90 tumor-specific genes were analyzed simultaneously on a 96-well plate using the Applied Biosystems 7500 Real-Time PCR (Applied Biosystems). The PCR program was initiated at 95°C for 10 min, followed by 40 thermal cycles, each at 95°C for 15 s and at 60°C for 1 min. For each sample, the turnaround time of Real-Time PCR analysis is 90 min.

### Data Analysis

Gene expression analysis was performed using R software and packages from the Bioconductor project (15–18). For each specimen, the expression profile of the 90 tumor-specific genes was analyzed, and a similarity score was obtained for each of the 21 tumor types on the test panel (14). The similarity score represents the degree of certainty by which the gene expression pattern of the specimen matches the gene expression pattern of the indicated tumor type, and scores range from 0 (low certainty) to 100 (high certainty) with a sum of 100 across all 21 tumor types on the panel.

For each specimen, the predicted primary site of the tumor was compared with the reference diagnosis. In the current study, a true positive result was indicated when the predicted tumor type was breast cancer. When the predicted tumor type and reference diagnosis did not match, the result for that specimen was marked as an error. The end point was diagnostic accuracy, defined as the number of correct predictions divided by the total number of evaluable cases.

A non-parametric analysis (rank product) was performed to identify genes that were differentially expressed between the 115 TNBC (73 primary site samples and 42 lymph node metastasis samples) and 188 non-TNBC samples. Gene expression data for the 188 non-TNBC samples were retrieved from a comprehensive cohort of FFPE tumor samples that was used to assess the overall performance of the 90-gene expression signature on 21 major tumor types (14). All non-TNBC samples were collected from Fudan University Shanghai Cancer Center. The clinical characteristic of non-TNBC samples were summarized in Table S1. Of note, the 188 non-TNBC samples did not overlap with any of the 115 TNBC samples validated in this study. Genes with an estimated percentage of false predictions (PFP) below 0.001 were selected as candidate markers for TNBC.

Discriminative power of the selected genes was assessed by hierarchical clustering and visualized using a two-dimensional heat map to examine separation between TNBC and non-TNBC tumors.

## Results

### Patients and Samples

All TNBC cases were confirmed as female patients. Cases were divided into three groups based on the biopsy site to include 42 paired primary breast tumors (PT) and 42 lymph node metastases (LNM), as well as 31 distant organ metastases (DOM). Table 1 shows detailed clinicopathological characteristics of the validation samples. The median age of patients at diagnosis was 51 years, ranging from 27 to 84 years. There were six early onset TNBC cases (≤35 years old) and sixty-seven late onset TNBC cases. Of 42 PT cases, 19 were on the left breast, and 23 were on the right breast. The most common histological subtype was invasive ductal carcinoma, while only one case was ductal carcinoma *in situ*. For DOM cases, lung, liver, and brain are the most common metastatic sites. We further investigated the family history of 73 patients. We found that five patients have a family history with breast cancer or ovarian cancer, and 14 patients have a family history with other neoplastic disorders, like colorectal cancer, gastric cancer, liver cancer and so on. Detailed clinical information for each patient is described in Table S2.

**Table 1:** Patients and tumors characteristics included in this study.

**Characteristic**	**Primary tumor/paired lymph node metastasis**	**Distant organ metastasis**
**No. patients**	42	31
**Age, years**		
Median	51	
Range	27–84	
**Pathological type**		
IDC^a^	41	31
Non-IDC	1	0
**Tumor laterality**		
Left	19	/
Right	23	/
**Metastatic sites**		
Liver	/	7
Lung	/	7
Brain	/	7
Head and neck	/	3
Lumbar vertebrae	/	2
Thoracic wall	/	1
Thoracic vertebrae	/	1
Humerus	/	1
Colorectum	/	1
Ovary	/	1

a *IDC, Invasive ductal carcinoma*.

### Performance of the 90-Gene Expression Signature in TNBC Primary Tumors, Lymph Node Metastases, and Distant Organ Metastases

Tissue sections from 115 samples were processed for isolation of total RNA, and concentrations ranged from 1.82 to 371.81 ng/μL, with a median of 63.23 ng/μL. The A260/A280 ratio ranged from 1.71 to 2.09.

**Figure 1:**
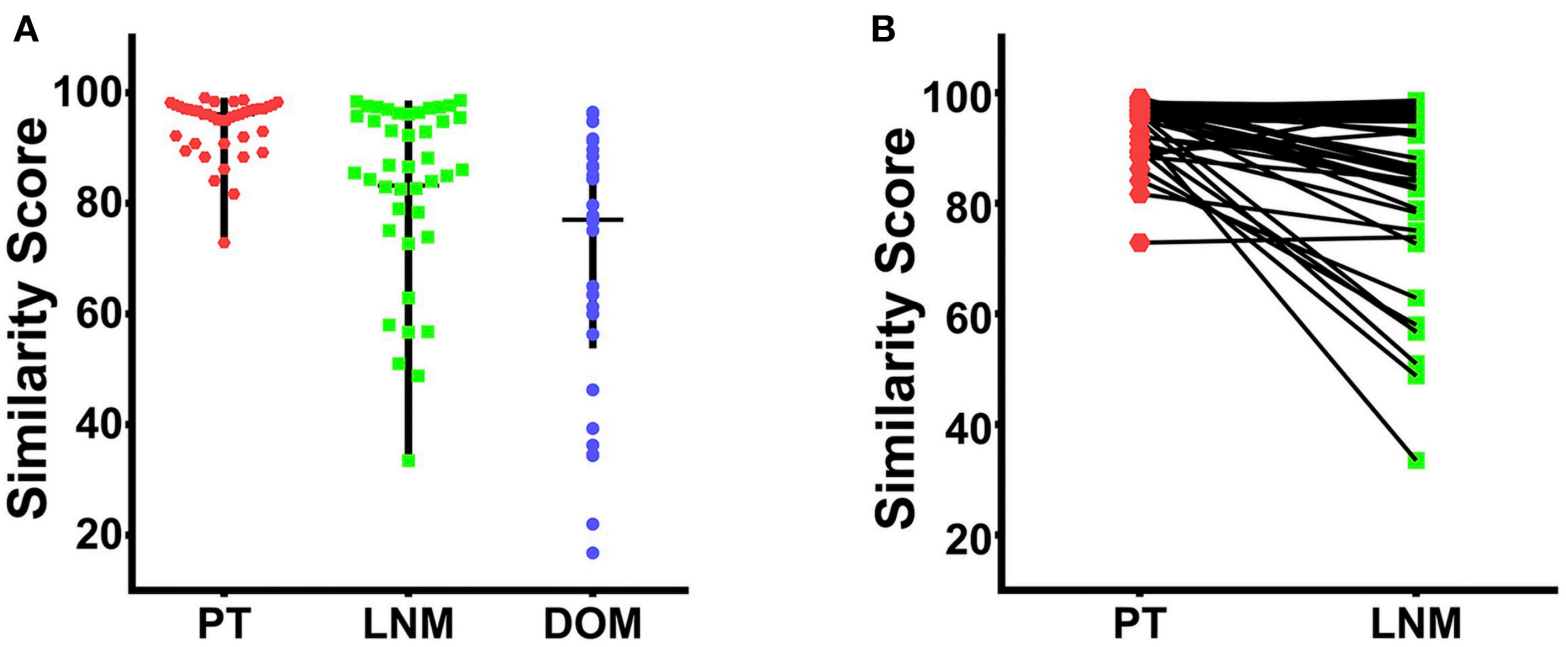
The similarity score of 115 cases of TNBC. **(A)** The distribution of similarity score for the PT (red dots), LNM (blue dots), and DOM (green dots) respectively. **(B)** The differences in the similarity score of 42 paired PT and LNM samples.

The 90-gene expression signature exhibited 97.4% agreement with the reference diagnosis (112/115, 95% confidence interval: 0.92–0.99). The concordance rate was 97.6% (41/42), 97.6% (41/42), and 96.8% (30/31) for PT, LNM, and DOM cases, respectively (Table 2). Distribution of the similarity scores for the three groups is shown in Figure 1A. For cases that were concordant with the reference diagnosis, the similarity score of PT cases ranged from 72.9 to 99.1, with a median similarity score of 96.1, whereas the similarity score of LNM cases ranged from 33.5 to 98.6, with a median similarity score of 86.6. The similarity score of cases that were discordant with the reference diagnosis was 31.4 and 65.7 for paired PT and LNM cases, respectively. Differences in the similarity score of paired samples are listed in Figure 1B. Next, we evaluated the biopsy site and prediction results for DOM cases. The 90-gene expression signature identified the correct tissue of origin for 30 of 31 samples. The median similarity score was 77.05, ranging from 16.8 to 96.5, for cases that were concordant with the reference diagnosis.

**Table 2:** Performance of 90-gene expression signature in TNBC.

**Tumor type**	***n***	**Agreement**	**Accuracy (%)**
Primary breast tumor	42	41	97.60
Lymph node metastasis	42	41	97.60
Distant organ metastasis	31	30	96.80
Total accuracy			97.40

A discrepancy analysis was then performed to determine the characteristics of the three cases that were discordant with the reference diagnosis (Table 3). In one case, PT and LNM cases from the same patient were predicted to be brain tumor and germ cell tumor, respectively. Another case that was discordant with the reference diagnosis was from a patient whose tumor was histopathologically diagnosed as TNBC that had metastasized to the lung but was identified as a germ cell tumor by the gene expression signature.

**Table 3:** Investigation of cases with discordant 90-gene expression signature results.

**Patient ID**	**Reference diagnosis**	**History**	**Immunohistochemical staining**	**90-gene expression signature results**	**Highest similarity score**
1	Primary breast cancer	51-year-old woman with right-sided breast cancer, 6 lymph nodes were removed, and 1 was determined to be positive.	ER (–), PR (–), HER2 (0), Ki67 (60%), GATA3 (–), GCDFP15 (–), Mammaglobin (–), CK5/6 (+)	Brain	31.4
	Lymph node metastasis of breast cancer		CK14 (–), EGFR (–)	Germ cell	65.7
2	Metastatic breast cancer to the lung	49-year-old women with invasive ductal breast cancer who received breast-conserving surgery 18 months before. Lymph nodes were negative. The patient eventually developed lung metastasis.	ER (–), PR (–), HER2 (0), GATA3 (–), GCDFP15 (–), Mammaglobin (–)	Germ cell	78

### Performance of the 90-Gene Expression Signature in Non-TNBC Metastatic Tumors

A total of 12 non-TNBC metastatic cases were included in the study. The clinical data of 12 patients were characterized in Table S3. The cohort included seven males and five females with a median age of 58.5 years, ranging from 34 to 76 years. The metastatic sites of non-TNBC tumors included lymph node (four cases), brain (four cases), liver (three cases), and colorectum (one case). Thus, the distribution of metastatic site of non-TNBC tumors was very similiar to the distribution of metastatic site of TNBC. For the 12 cases, predictions of 90-gene expression signature showed 100% concordance rate with the reference diagnosis of non-TNBC metastatic tumors.

### Specific Patient Examples

**Case 1**. A 50-year-old woman noticed masses in her left axilla and left supraclavicular area. She underwent biopsies of supraclavicular masses in another hospital and was diagnosed with poorly differentiated carcinoma (PDC) that was Villin positive. She subsequently received six cycles of chemotherapy as digestive tract tumors and exhibited cancer progression. The gene expression profile indicated that the metastases were more likely to originate from breast carcinoma. Subsequently pathology consultation showed PDC (IHC stains: AE1/AE3+, ER–, PR–, HER-2 0, GCDFP-15–, TTF-1–, and PAX8–). Tumors were subsequently controlled after changing to a regimen specific for TNBC. During follow up 15 months later, space-occupying lesions in the left breast were found, and a core needle biopsy revealed invasive breast cancer.

**Comment**. The patient had breast cancer presenting 15 months after her presentation with metastatic carcinoma; therefore, the diagnosis can only depend upon IHC. As IHC stains revealed ER–, PR–, HER−2 0, it is difficult to identify triple-negative breast cancer in metastatic cancer without a prior history of breast cancer. The 90-gene expression profiling of her initial biopsy predicted breast carcinoma.

**Case 2**. A 47-year-old woman noticed masses in her left lower neck and supraclavicular area. Ultrasound of the breast showed adenosis. However, no other space occupying lesions were identified by PET-CT. Pathology revealed squamous cell carcinoma (IHC stains: ER–, PR–, HER−2 0). The gene expression profile indicated breast cancer. Ultrasounds of the thyroid, breast, collarbone, and neck and axillary lymph nodes were added and showed a 22 mm ^*^10.5 mm mixed echo focus in the left breast (BI-RADS 4C).

**Comment**. This patient has triple negative breast cancer that was confirmed by imaging examination of the breast. The imaging test and IHC stains were primarily non-diagnostic, and the 90-gene expression profiling of her initial biopsy predicted breast carcinoma, highlighting the organ that needed to be inspected.

### Identification of Novel TNBC Biomarkers

**Figure 2:**
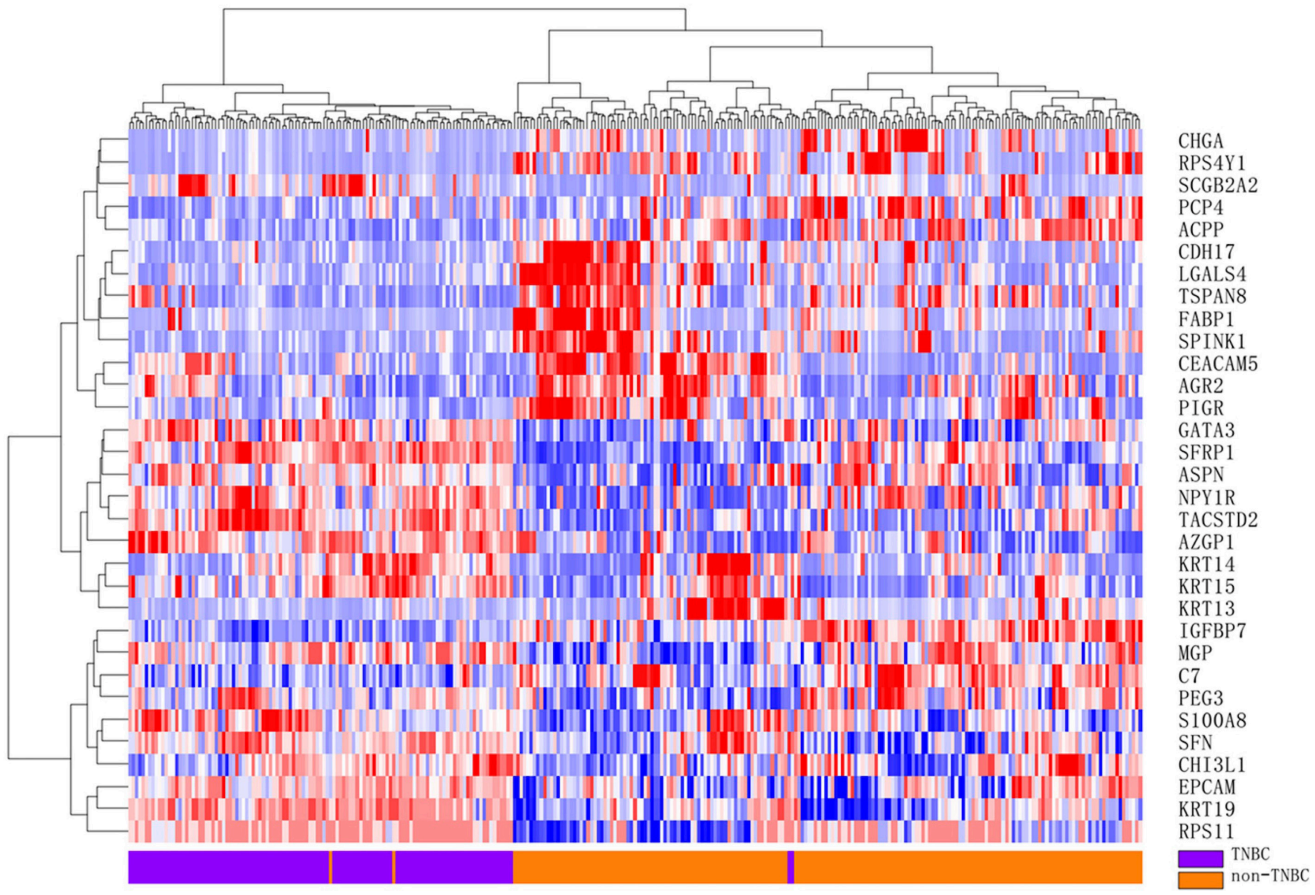
Hierarchical clustering analysis of 32 differentially gene expression between 115 TNBC and 188 non-TNBC samples. Normalized gene expression intensities were shifted to mean = 0, and rescaled to STD = 1 to enhance the expression differences. The average linkage hierarchical clustering method was performed where the metric of similarity was Pearson's correlation between every pair of samples. The right panel indicates the official symbol of 32 genes. The left panel shows a dendrogram of hierarchical clustering of these genes. Colored pixels capture the magnitude of the expression for any gene, where shades of red and blue represent over-expression and under-expression, respectively, relative to the mean for each gene. The upper panel shows a dendrogram of hierarchical clustering of samples. The histological type of each sample is indicated in the bottom panel, with TNBC tumor samples shown in purple and non-TNBC tumor samples in orange. The samples clustered into two groups that closely follow the tumor types.

Furthermore, rank product analysis was performed to select a small subset of genes from the 90-gene panel with diagnostic utility for TNBC. The top 17 upregulated genes and 15 downregulated genes with PFP below 0.001 were identified as candidate genes to distinguish TNBC tumors from other types of tumors. These genes are described in more detail in Table 4. Hierarchical clustering based on the 32 differentially expressed genes showed clear separation between 115 TNBC and 188 non-TNBC tumors, indicating excellent discriminative power of the selected candidate markers (Figure 2).

**Table 4:** Description of 17 up-regulated and 15 down-regulated genes in triple-negative breast cancer.

**Symbol**	**Description**	**Cytoband**	**Regulation (TNBC/non-TNBC)**	**Fold change**	***P*-value**
AZGP1	Alpha-2-glycoprotein 1, zinc-binding	7q22.1	Up	2.3	1.40E-35
KRT19	Keratin 19	17q21.2	Up	2.3	1.80E-33
RPS11	Ribosomal protein S11	19q13.3	Up	2.1	9.80E-29
SFRP1	Secreted frizzled related protein 1	8p11.21	Up	1.9	6.10E-19
EPCAM	Epithelial cell adhesion molecule	2p21	Up	1.9	8.50E-19
TACSTD2	Tumor-associated calcium signal transducer 2	1p32	Up	1.8	9.70E-17
MGP	Matrix Gla protein	12p12.3	Up	1.6	3.60E-12
SFN	Stratifin	1p36.11	Up	1.6	8.80E-11
GATA3	GATA binding protein 3	10p15	Up	1.6	8.80E-11
S100A8	S100 calcium binding protein A8	1q21	Up	1.5	2.20E-09
CHI3L1	Chitinase 3 like 1	1q32.1	Up	1.4	5.90E-07
KRT15	Keratin 15	17q21.2	Up	1.4	1.20E-06
KRT14	Keratin 14	17q21.2	Up	1.3	8.00E-06
NPY1R	Neuropeptide Y receptor Y1	4q32.2	Up	1.4	1.10E-05
ASPN	Asporin	9q22	Up	1.3	5.50E-04
PEG3	Paternally expressed 3	19q13.4	Up	1.3	7.00E-04
SCGB2A2	Secretoglobin family 2A member 2	11q13	Up	1.3	8.50E-04
SPINK1	Serine peptidase inhibitor, Kazal type1	5q32	Down	0.6	1.50E-12
PIGR	Polymeric immunoglobulin receptor	1q31-q41	Down	0.6	1.20E-10
KRT13	Keratin 13	17q21.2	Down	0.6	5.40E-09
RPS4Y1	Ribosomal protein S4, Y-linked 1	Yp11.3	Down	0.6	7.80E-09
CHGA	Chromogranin A	14q32	Down	0.6	3.80E-08
FABP1	Fatty acid binding protein 1	2p11	Down	0.7	4.90E-08
TSPAN8	Tetraspanin 8	12q21.1	Down	0.7	1.20E-07
ACPP	Acid phosphatase, prostate	3q22.1	Down	0.7	8.70E-07
LGALS4	Galectin 4	19q13.2	Down	0.7	3.30E-06
CDH17	Cadherin 17	8q22.1	Down	0.7	9.80E-06
PCP4	Purkinje cell protein 4	21q22.2	Down	0.7	2.20E-05
C7	Complement C7	5p13	Down	0.7	4.00E-04
AGR2	Anterior gradient 2, protein disulphide isomerase family member	7p21.3	Down	0.8	4.10E-04
IGFBP7	Insulin like growth factor binding protein 7	4q12	Down	0.7	6.20E-04
CEACAM5	Carcinoembryonic antigen related cell adhesion molecule 5	19q13.1-q13.2	Down	0.8	7.00E-04

## Discussion

In this study, we evaluated the potential usefulness of a 90-gene expression signature to identify TNBC using FFPE samples. Among all samples, 97.6% of PT (41/42) and LNM (41/42) and 96.8% of DOM (30/31) were correctly classified. Among the 19 patients have a family history with breast cancer or other neoplastic disorders, one with family history of colorectal cancer was misclassified. Furthermore, the 90-gene expression signature achieved 100% accuracy in six early onset patients and 97% accuracy in sixty-seven late onset patients. According to the results, there was no significant correlation between age, family history and signature performance. The overall accuracy of 97.4% reported here indicates excellent performance of the 90-gene expression signature in the identification of tumor origin in a heterogeneous group of TNBC tumors.

For the first time in 2006, Bryan et al. explicitly defined TNBC based on negative expression of ER, PR and HER-2 (19). Although TNBC has been extensively studied in clinical and pre-clinical settings, there are only a few reports on suitable differential diagnostics for this subtype of breast cancer. The standard IHC markers employed in most pathology laboratories are useful for identifying breast cancer; however, clinical identification of TNBC, in particular for metastatic tumors without a prior history of breast cancer, is difficult and thus urgently needed. Notably, in most cases, the treatment of choice is dictated by the differential diagnosis. For example, in the case of a small, solitary lung tumor in the absence of swollen lymph nodes, the patient can be treated with either chemotherapy or partial resection of the lung when the lung tumor is diagnosed as metastatic breast cancer. However, when the same tumor is diagnosed as primary non-small cell lung cancer, standard lobectomy may be the preferred treatment (9). Recently, the IMpassion130 trial showed that for patients with PD-L1-positive tumors, the combination of atezolizumab and nab-paclitaxel led to a significant improvement in overall survival of 25.0 vs. 15.5 months with nabpaclitaxel and placebo. Therefore, precise diagnosis of TNBC is more important than ever for clinical decision making in the era of immunotherapy (5). The results reported here demonstrate that the 90-gene expression signature reliably identifies TNBC in both primary and metastatic settings. To the best of our knowledge, this is the first report of a novel molecular assay that can be used to differentially diagnose TNBC.

Two specific patient examples were discussed to some extent to illustrate the pathologic and clinical significance of our 90-gene expression signature. For the first case, histopathologically, a Villin+ lesion would be excluded from a diagnosis of breast cancer, and the diagnosis will be leading to the incorrect orientation if the pathologist completely relies upon this marker when imaging analysis showed no positive result. The molecular profile assay provides an effective approach to avoid current limitations of IHC, including inevitably low specificity, false positives, and lack of an accurate molecule biomarker for tumor origin. Clinically, in patients with highly suspected breast cancer, use of a molecular profile assay may be able to identify tumor attributes more quickly when imaging and IHC examination are ineffective. For the second case, the use of a molecular profile assay not only provided evidence for further selection of imaging examination methods but also provided suggestions on which part of the body needed to be examined.

Those three cases with discordant 90-gene expression signature results revealed an intriguing topic: how can TNBC be recognized as a brain or germ cell original tumor? Embryologically, both breast and brain develop from the outer layer of the ovule; therefore, it is understandable that tumors from these two organs may show high similarity in their gene profiles; on the other hand, it has been reported that basal-like breast cancers can exhibit high mRNA expression correlations with serous ovarian cancers and lung squamous carcinomas, suggesting that tumors from different organs may share the same driving events for carcinogenesis (20). Further endeavors should be focus on how to overcome this obstacle.

Notably, 32 of the 90 genes in the panel were significantly differentially expressed between TNBC and non-TNBC tumors. Among these genes, the 17 genes upregulated in TNBCs are particularly interesting. Several of these genes are associated with TNBC. In a recent study by Lehmann et al. (21) *KRT14* and *KRT19* were found to be differentially expressed between basal-like and luminal-like TNBC. Expression of *SFRP1* was found to be significantly higher in TNBC than in other breast cancer subtypes. Additionally, *SFRP1* expression is significantly correlated with an increased probability of a positive response to neoadjuvant chemotherapy (22). Moreover, expression of *KRT15* was found to be 3.8- and 3.5-fold higher in mammary stem cells than in myoepithelial cells and luminal cells of TNBC tumors, respectively (23). Regarding the remaining differentially upregulated genes, such as *AZGP1, PIGR, SPINK1, RPS11, TACSTD2*, and *EPCAM*, we are the first to report their overexpression in TNBC. Future studies on the proteins encoded by these genes may provide useful insights into potential novel markers for differential diagnosis of TNBC.

In conclusion, this 90-gene expression signature shows high accuracy in identifying primary and metastatic TNBC tumors, suggesting the potential of this 90-gene expression signature as a complementary tool to support the diagnosis of TNBC. In clinical practice, common sites of breast cancer metastasis are bone, lung, and liver. When there is a solitary mass in these organs, it is extremely important to differentiate whether it is a metastasis from breast cancer or a second primary. In cases where the morphology and immunohistochemistry work-up cannot confirm the primary (most often in the TNBC setting), the 90-gene expression signature could provide valuable information for differential diagnosis. Furthermore, this molecular biomarker may also be useful for distinguishing primary TNBC tumors from other poorly differentiated tumors from rare tissue origin that metastasize to the breast, especially in the absence of an *in-situ* component in needle biopsy samples.

## Ethics Statement

This study was carried out in accordance with the recommendations of the institutional review board of Fudan University Shanghai Cancer Center; Zhejiang Cancer Hospital; The First Affiliated Hospital of Wenzhou Medical University; The First People's Hospital of Changzhou; The Third Affiliated Hospital of Soochow University; Changzhou No. 2 People's Hospital; the Affiliated Hospital of Nanjing Medical University and The Affiliated Hospital of Jiangnan University with written informed consent from all subjects. All subjects gave written informed consent in accordance with the Declaration of Helsinki. The protocol was approved by the institutional review board of Fudan University Shanghai Cancer Center; Zhejiang Cancer Hospital; The First Affiliated Hospital of Wenzhou Medical University; The First People's Hospital of Changzhou; The Third Affiliated Hospital of Soochow University; Changzhou No. 2 People's Hospital; the Affiliated Hospital of Nanjing Medical University and The Affiliated Hospital of Jiangnan University.

## Author Contributions

QW and QX participated in the study design and data analysis, and draft the manuscript. MX carried out the immunoassays and help to draft the manuscript. JC, CC, CQ, and YS performed molecular biological experiments. YC, LC, QX, XD, and WY conceived the study, participated in its design and coordination and help to draft the manuscript. All authors read and approved the final manuscript.

### Conflict of Interest Statement

JC, CC, CQ, YS, and QX are employees of Canhelp Genomics. The remaining authors declare that the research was conducted in the absence of any commercial or financial relationships that could be construed as a potential conflict of interest.

## References

[1] Chen W Zheng R Baade PD Zhang S Zeng H Bray F. Cancer statistics in China, 2015. Cancer J Clin. (2016) 66:115–32. 10.3322/caac.21338 26808342

[2] Zhang J Wang Z Hu X Wang B Wang L Yang W. Cisplatin and gemcitabine as the first line therapy in metastatic triple negative breast cancer. Int J Cancer. (2014) 136:204–11. 10.1002/ijc.28966 24824628

[3] Lehmann BD Pietenpol JA. Identification and use of biomarkers in treatment strategies for triple-negative breast cancer subtypes. J Pathol. (2013) 232:142–50. 10.1002/path.4280 24114677 PMC4090031

[4] Penault-Llorca F Viale G. Pathological and molecular diagnosis of triple-negative breast cancer: a clinical perspective. Ann Oncol. (2012) 23:vi19–22. 10.1093/annonc/mds190 23012297

[5] Schmid P Adams S Rugo HS Schneeweiss A Barrios CH Iwata H Atezolizumab and Nab-paclitaxel in advanced triple-negative breast cancer. N Engl J Med. (2018) 376:2108–21. 10.1056/NEJMoa1809615 30345906

[6] Ordó-ez NG Value of GATA3 immunostaining in tumor diagnosis: a review. Adv Anat Pathol. (2013) 20:352–60. 10.1097/PAP.0b013e3182a28a68 23939152

[7] Krings G Nystrom M Mehdi I Cohra P Chen Y. Diagnostic utility and sensitivities of GATA3 antibodies in triple-negative breast cancer. Hum Pathol. (2014) 45:2225–32. 10.1016/j.humpath.2014.06.022 25150746

[8] Huo L Zhang J Gilcrease MZ Gong Y Wu Y Zhang H. Gross cystic disease fluid protein-15 and mammaglobin A expression determined by immunohistochemistry is of limited utility in triple-negative breast cancer. Histopathology. (2012) 62:267–74. 10.1111/j.1365-2559.2012.04344.x 22963676 PMC3881372

[9] Sasaki E Tsunoda N Hatanaka Y Mori N Iwata H Yatabe Y. Breast-specific expression of MGB1/mammaglobin: an examination of 480 tumors from various organs and clinicopathological analysis of MGB1-positive breast cancers. Mod Pathol. (2006) 20:208–14. 10.1038/modpathol.3800731 17192791

[10] Kerr SE Schnabel CA Sullivan PS Zhang Y Singh V Carey B. Multisite validation study to determine performance characteristics of a 92-gene molecular cancer classifier. Clin Cancer Res. (2012) 18:3952–60. 10.1158/1078-0432.CCR-12-0920 22648269

[11] Kerr SE Schnabel CA Sullivan PS Zhang Y Huang VJ Erlander MG. A 92-gene cancer classifier predicts the site of origin for neuroendocrine tumors. Mod Pathol. (2013) 27:44–54. 10.1038/modpathol.2013.105 23846576

[12] Benjamin H Lebanony D Rosenwald S Cohen L Gibori H Barabash N. A diagnostic assay based on microRNA expression accurately identifies malignant pleural mesothelioma. J Mol Diagn. (2010) 12:771–9. 10.2353/jmoldx.2010.090169 20864637 PMC2963911

[13] Xu Q Chen J Ni S Tan C Xu M Dong L. Pan-cancer transcriptome analysis reveals a gene expression signature for the identification of tumor tissue origin. Mod Pathol. (2016) 29:546–56. 10.1038/modpathol.2016.60 26990976

[14] Ye Q Wang Q Qi P Chen J Ren W Xu M Development and validation of a 90-gene real-time PCR assay for tumor origin identification. In: WIN 2018 ABSTRACT BOOK. (2018): P5.4. Available online at: https://www.eiseverywhere.com/docs/4545/225884

[15] Ihaka R Gentleman R R: a language for data analysis and graphics. arXiv (1996) 5:299–314. 10.1080/10618600.1996.10474713

[16] Gentleman RC Carey VJ Bates DM Bolstad B Dettling M Dudoit S. Bioconductor: open software development for computational biology and bioinformatics. Genome Biol. (2004) 5:R80–16. 10.1186/gb-2004-5-10-r80 15461798 PMC545600

[17] Hong F Breitling R McEntee CW Wittner BS Nemhauser JL Chory J. RankProd: a bioconductor package for detecting differentially expressed genes in meta-analysis. Bioinformatics. (2006) 22:2825–7. 10.1093/bioinformatics/btl476 16982708

[18] Simon R Lam A Li M-C Ngan M Menenzes S Zhao Y Analysis of gene expression data using BRB-array tools. Cancer Inform. (2007) 3:11–7. 10.1177/117693510700300022 19455231 PMC2675854

[19] Bryan BB Schnitt SJ Collins LC. Ductal carcinoma *in situ* with basal-like phenotype: a possible precursor to invasive basal-like breast cancer. Mod Pathol. (2006) 19:617–21. 10.1038/modpathol.3800570 16528377

[20] Cancer Genome Atlas Network Comprehensive molecular portraits of human breast tumours. Nature. (2012) 490:61–70. 10.1038/nature11412 23000897 PMC3465532

[21] Lehmann BD Bauer JA Chen X Sanders ME Chakravarthy AB Shyr Y. Identification of human triple-negative breast cancer subtypes and preclinical models for selection of targeted therapies. J Clin Invest. (2011) 121:2750–67. 10.1172/JCI45014 21633166 PMC3127435

[22] Bernemann C Hülsewig C Ruckert C Schäfer S Blümel L Hempel G Influence of secreted frizzled receptor protein 1 (SFRP1) on neoadjuvant chemotherapy in triple negative breast cancer does not rely on WNT signaling. Mol Cancer. (2014) 13:174–12. 10.1186/1476-4598-13-174 25033833 PMC4110378

[23] Soady KJ Kendrick H Gao Q Tutt A Zvelebil M Ordonez LD. Mouse mammary stem cells express prognostic markers for triple-negative breast cancer. Br Cancer Res. (2015) 17:31. 10.1186/s13058-015-0539-6 25849541 PMC4381533

