# Supplementary figures and images for: Gene Expression Profiling for Diagnosis of Triple-Negative Breast Cancer: A Multicenter, Retrospective Cohort Study
Source: Front Oncol. 2019 May 7;9:354. doi: 10.3389/fonc.2019.00354 (PMC6513966; doi:10.3389/fonc.2019.00354)

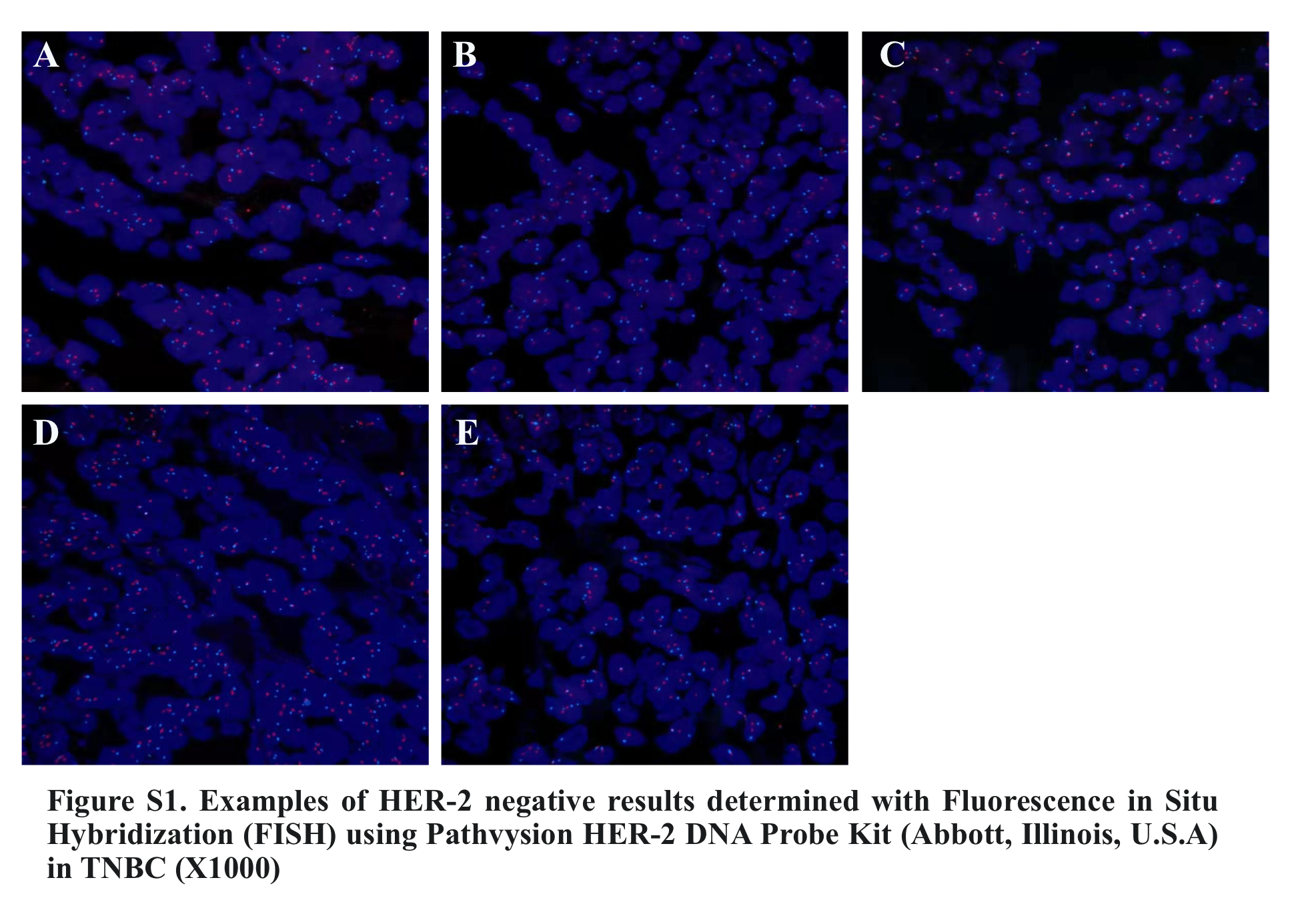

Supplement: Supplementary file 4 [file Image_1.PNG]
